# Comparison of various molecular methods for rapid differentiation of intestinal bifidobacteria at the species, subspecies and strain level

**DOI:** 10.1186/s12866-016-0779-3

**Published:** 2016-07-22

**Authors:** Piotr Jarocki, Marcin Podleśny, Elwira Komoń-Janczara, Jagoda Kucharska, Agnieszka Glibowska, Zdzisław Targoński

**Affiliations:** Department of Biotechnology, Human Nutrition and Food Commodities, University of Life Sciences in Lublin, 8 Skromna St., 20-704 Lublin, Poland

**Keywords:** *Bifidobacterium*, Differentiation, ARDRA, RAPD, rep-PCR, SDS-PAGE fingerprinting

## Abstract

**Background:**

Members of the genus *Bifidobacterium* are anaerobic Gram-positive *Actinobacteria*, which are natural inhabitants of human and animal gastrointestinal tract. Certain bifidobacteria are frequently used as food additives and probiotic pharmaceuticals, because of their various health-promoting properties.

Due to the enormous demand on probiotic bacteria, manufacture of high-quality products containing living microorganisms requires rapid and accurate identification of specific bacteria. Additionally, isolation of new industrial bacteria from various environments may lead to multiple isolations of the same strain, therefore, it is important to apply rapid, low-cost and effective procedures differentiating bifidobacteria at the intra-species level. The identification of new isolates using microbiological and biochemical methods is difficult, but the accurate characterization of isolated strains may be achieved using a polyphasic approach that includes classical phenotypic methods and molecular procedures. However, some of these procedures are time-consuming and cumbersome, particularly when a large group of new isolates is typed, while some other approaches may have too low discriminatory power to distinguish closely related isolates obtained from similar sources.

**Results:**

This work presents the evaluation of the discriminatory power of four molecular methods (ARDRA, RAPD-PCR, rep-PCR and SDS-PAGE fingerprinting) that are extensively used for fast differentiation of bifidobacteria up to the strain level. Our experiments included 17 reference strains and showed that in comparison to ARDRA, genotypic fingerprinting procedures (RAPD and rep-PCR) seemed to be less reproducible, however, they allowed to differentiate the tested microorganisms even at the intra-species level. In general, RAPD and rep-PCR have similar discriminatory power, though, in some instances more than one oligonucleotide needs to be used in random amplified polymorphic DNA analysis. Moreover, the results also demonstrated a high discriminatory power of SDS-PAGE fingerprinting of whole-cell proteins. On the other hand, the protein profiles obtained were rather complex, and therefore, difficult to analyze.

**Conclusions:**

Among the tested procedures, rep-PCR proved to be the most effective and reliable method allowing rapid differentiation of *Bifidobacterium* strains. Additionally, the use of the BOXA1R primer in the differentiation of 21 *Bifidobacterium* strains, newly isolated from infant feces, demonstrated slightly better discriminatory power in comparison to PCR reactions with the (GTG)_5_ oligonucleotide. Thus, BOX-PCR turned out to be the most appropriate and convenient molecular technique in differentiating *Bifidobacterium* strains at all taxonomic levels.

**Electronic supplementary material:**

The online version of this article (doi:10.1186/s12866-016-0779-3) contains supplementary material, which is available to authorized users.

## Background

It was estimated that about 3 % of bacteria that are normal inhabitants of human gastrointestinal tract belong to the genus *Bifidobacterium* [1]. Previous research supported by clinical trials showed that some metabolic activities of these microbiota may lead to numerous health benefits for the host [2]. It was documented that members of the genus *Bifidobacterium* may enhance lactose tolerance, reduce the effects of diarrhea and constipation and prevent diseases by inhibiting intestinal colonization by pathogenic bacteria [3–6]. It was also reported that bifidobacteria may have anti-carcinogenic properties, play an important role in immunomodulation and even decrease serum cholesterol level [7–9]. Therefore, these bacteria are commonly used as probiotics in fermented foods and dietary supplements.

The increasing application of bifidobacteria as health-benefit ingredients of high-quality foods and pharmaceuticals requires rapid and accurate identification of these microorganisms at the species, subspecies and even strain level. Unambiguous and reliable identification of such isolates is problematic using microbiological and biochemical methods owing to their low discriminatory power. Therefore, a polyphasic procedure, involving a combination of classical phenotypic methods and molecular techniques can provide more accurate and reliable characterization of the isolates [10, 11].

Previous studies used various molecular methods to differentiate and specifically identify *Bifidobacterium* strains [12, 13]. Among them, a sequence analysis of 16S rRNA has been widely used for both preparation of species-specific PCR primers and bacterial phylogeny analysis [14, 15]. However, some bifidobacterial species showed a high degree of similarity in this gene sequence, thus alternative molecular techniques were applied, such as multilocus sequence analysis (MLSA), randomly amplified polymorphic DNA (RAPD), amplified-fragment length polymorphism (AFLP), ribotyping, rep-PCR, RFLP, pulsed-field gel electrophoresis (PFGE) or SDS-PAGE of whole-cell proteins [12, 16–21]. Some of these procedures are arduous and time-consuming, especially when a large group of isolates is considered. Yet, some other approaches may have a too low discriminatory power to distinguish between closely related isolates from similar environments. In addition, most of the previous studies were carried out using different bacterial strains and different methodology, therefore, it is very hard to select the most reliable and convenient method for rapid differentiation of bifidobacterial strains.

The main aim of this work was to compare the discriminatory power of four molecular methods commonly applied as a rapid tool identifying bifidobacteria at all taxonomic levels. Furthermore, new isolates from child feces were differentiated using the selected, most effective procedures.

## Methods

### Bacterial strains and culture conditions

*Bifidobacterium* strains used in this study Table 1 were obtained from the German Collection of Microorganisms (DSMZ) and Agricultural Research Service Culture Collection (NRRL – Northern Regional Research Laboratory). Additionally, 21 new isolates from child feces were also used. All strains were cultivated in the modified Garche’s medium containing peptone, 20 g/l; yeast extract, 2 g/l; lactose, 10 g/l; L-cysteine hydrochloride, 0.4 g/l; sodium acetate, 6 g/l; MgSO_4_x7H_2_O, 0.12 g/l; KH_2_PO_4_, 2 g/l; Na_2_HPO_4_x12 H_2_O, 2.5 g/l; pH 6.4 [22]. The cultures were incubated anaerobically at 37 °C using anaerobic jars and AnaeroGen sachets (Oxoid, Basingstoke, UK) for 24 or 48 h.

**Table 1:** List of reference bifidobacterial strains used in this study

Strain	
*Bifidobacterium adolescentis* DSM 20083^T^	
*Bifidobacterium adolescentis* DSM 20086	
*Bifidobacterium adolescentis* DSM 20087	
*Bifidobacterium animalis* subsp. *lactis* NRRL B-41405	
*Bifidobacterium animalis* subps. *animalis* NRRL B-41406^T^	
*Bifidobacterium bifidum* DSM 20456^T^	
*Bifidobacterium breve* DSM 20091	
*Bifidobacterium breve* NRRL B-41408^T^	
*Bifidobacterium catenulatum* DSM 20224	
*Bifidobacterium longum* subsp. *infantis* ATCC 15697^T^	
*Bifidobacterium longum* subsp. *longum* NRRL B-41409^T^	
*Bifidobacterium longum* subsp. *suis* NRRL B-41407^T^	
*Bifidobacterium pseudocatenulatum* DSM 20439	
*Bifidobacterium pseudolongum* subsp. *globosum* DSM 20092^T^	
*Bifidobacterium pseudolongum* subsp. *pseudolongum* DSM 20094	
*Bifidobacterium pseudolongum* subsp. *pseudolongum* DSM 20095	
*Bifidobacterium pseudolongum* subsp. *pseudolongum* DSM 20099^T^	

### Total DNA preparation

For each strain, DNA extraction was performed from a 3-ml aliquot of 24-h culture [23]. After centrifugation, the supernatant was removed and cells were resuspended in 380 μl of 6.7 % (w/v) sucrose, 50 mM Tris-1 mM EDTA (pH 8.0) buffer. Next, 100 μl of a 50 mg/l lysozyme solution (MP Biomedicals, Santa Ana, USA) and 20 μl of mutanolysin (5 U/μl) (Sigma-Aldrich, Saint Louis, USA) were added and then the samples were incubated at 37 °C for 1 h. After incubation, 50 μl of 0.25 M EDTA, 50 mM Tris (pH 8.0) was added, and the cells were then treated with 30 μl of 20 % (w/v) sodium dodecyl sulfate, 50 mM Tris, 20 mM EDTA (pH 8.0). In addition, the proteins were digested by adding 20 μl of proteinase K (20 mg/ml) (Thermo Fisher Scientific, Waltham, USA) and incubated for 1 h at 50 °C.

Afterwards, DNA was purified from cell debris using a standard phenol-chloroform extraction method. Finally, the concentration of nucleic acid samples was measured using a NanoDrop spectrophotometer (Thermo Fisher Scientific, Waltham, USA).

### Restriction fragment length polymorphism of 16S rRNA genes

At the first stage, a specific fragment of the 16S rRNA gene was amplified using genus-specific primers, lm26 (5’- GATTCTGGCTCAGGATGAACG-3’) and lm3 (5’-CGGGTGCTICCCACTTTCATG-3’) [24], for each strain used in this study. The PCR reaction was performed in a 50-μl solution containing 2 U of DreamTaq DNA polymerase (Thermo Fisher Scientific, Waltham, USA), 300 μM of each deoxynucleoside triphosphate, PCR buffer (Thermo Fisher Scientific, Waltham, USA), 1 μM of each primer and 2 μl (~100 ng) of bacterial DNA. Amplification was carried out using the LabCycler (SensoQuest, Göttingen, Germany) programmed as follows: 4 min at 94 °C for initial denaturation and 35 cycles of 1 min at 94 °C, an annealing step at 57 °C for 3 min, 4 min at 72 °C for extension and 10 min at 72 °C for the final extension. After amplification, reaction mixtures were cooled down to 4 °C, and then frozen at −20 °C until further analysis. Next, 10 μl of the amplified DNA were digested using AluI, HaeIII and HinfI (Thermo Fisher Scientific, Waltham, USA) according to the manufacturer’s recommendations.

### Random amplification of polymorphic DNA (RAPD)

The RAPD analysis was performed with two different random primers PER1 (5’-AAGAGCCCG T-3’) [25] and CORR1 (5’-TGCTCTGCCC-3’) [26], using only one primer in a single PCR reaction. Template DNA (100 ng) and primers (final concentration of 1 μM each) were added to a 20-μl reaction mixture containing 200 μM of each deoxynucleoside triphosphate, 1 U (for RAPD with primer PER1) and 3 U (for reaction with CORR1) of *Taq* DNA polymerase and PCR buffer (Thermo Fisher Scientific, Waltham, USA). Amplifications were performed with initial template denaturation at 94 °C for 5 min, followed by 35 cycles of 1 min at 94 °C, an annealing step of 1 min at 36 °C and extension for 2 min at 72 °C, with the final extension for 10 min at 72 °C. The results obtained were analyzed by agarose gel electrophoresis as described below.

### PCR amplification of repetitive bacterial DNA elements (rep-PCR)

Two rep-PCR oligonucleotide primers were evaluated in this work: BOXA1R (5’-CTACGGCAAGGCGACGCTGACG-3’) and (GTG)_5_ (5’-GTGGTGGTGGTGGTG-3’). PCR was carried out in the total volume of 20 μl of the reaction mixture containing 1 U (for BOXA1R-PCR) and 2 U (for (GTG)_5_-PCR) of *Taq* DNA polymerase, 200 μM of each deoxynucleoside triphosphate, 1 μM of each primer, 50 ng of bacterial DNA and PCR buffer (Thermo Fisher Scientific, Waltham, USA). The amplification was conducted with template denaturation at 94 °C for 4 min, followed by 35 cycles (denaturation for 1 min at 94 °C, annealing for 1 min at 40 °C for BOXA1R-PCR or 50 °C for (GTG)_5_-PCR and extension for 2 min at 72 °C) and the final extension for 10 min at 72 °C. The PCR products were separated under standard conditions.

### SDS-PAGE fingerprinting

For cell-free extracts preparation, bifidobacteria were grown in 10 ml of Garche’s medium for 48 h. Then, the cells were harvested by centrifugation at 7142 × g for 10 min at 4 °C. The cell pellet was washed twice in PBS buffer and resuspended in 500 μl of 0.1 M sodium-phosphate buffer (pH 7.0). The cells were disrupted by sonication for 5 min with constant cooling, followed by centrifugation at 16,100 × g for 10 min at 4 °C. The supernatant was stored at −20 °C. Protein concentrations were measured by Bradford method [27], and bovine serum albumin was used as a standard. Next, samples (~10 μg) were tested by SDS-PAGE.

### Electrophoresis and data analysis

PCR amplicons and digestion products were resolved by agarose gel electrophoresis with 1.4 % (w/v) agarose in a Tris-acetate-EDTA buffer (TAE). The gels were stained with ethidium bromide (0.5 μg/ml) and visualized under UV light (GelDoc, Biorad, Hercules, USA). The protein samples were separated using a stacking gel containing 4 % acrylamide and 10 % resolving gel by the method of Laemmli [28]. Proteins were visualized by staining with Coomassie Brilliant Blue R-250. At least two replicates of each experiment were performed with little variation (only one example is shown). The results were analyzed using the Quantity One 1-D Analysis System (Biorad, Hercules, USA). Genetic distance between the isolates was calculated using the Dice coefficient of similarity, and then the strains were clustered in a dendrogram using the unweighted pair group method with arithmetic mean (UPGMA) analysis (data not shown).

## Results and discussion

### Amplified ribosomal DNA restriction analysis (ARDRA)

It is well documented that the 16S rRNA gene sequence analysis is a very useful molecular method for identification and phylogenetic analysis of prokaryotes [29, 30]. The sequence of the 16S rDNA gene has also been widely used in both taxonomy of the genus *Bifidobacterium* and preparation of species-specific PCR primers for accurate detection of microorganisms belonging to this important group of human intestinal microbiota [15, 31]. In addition, some researchers proved the applicability of ARDRA in *Bifidobacterium* species identification [32–34].

**Fig. 1:**
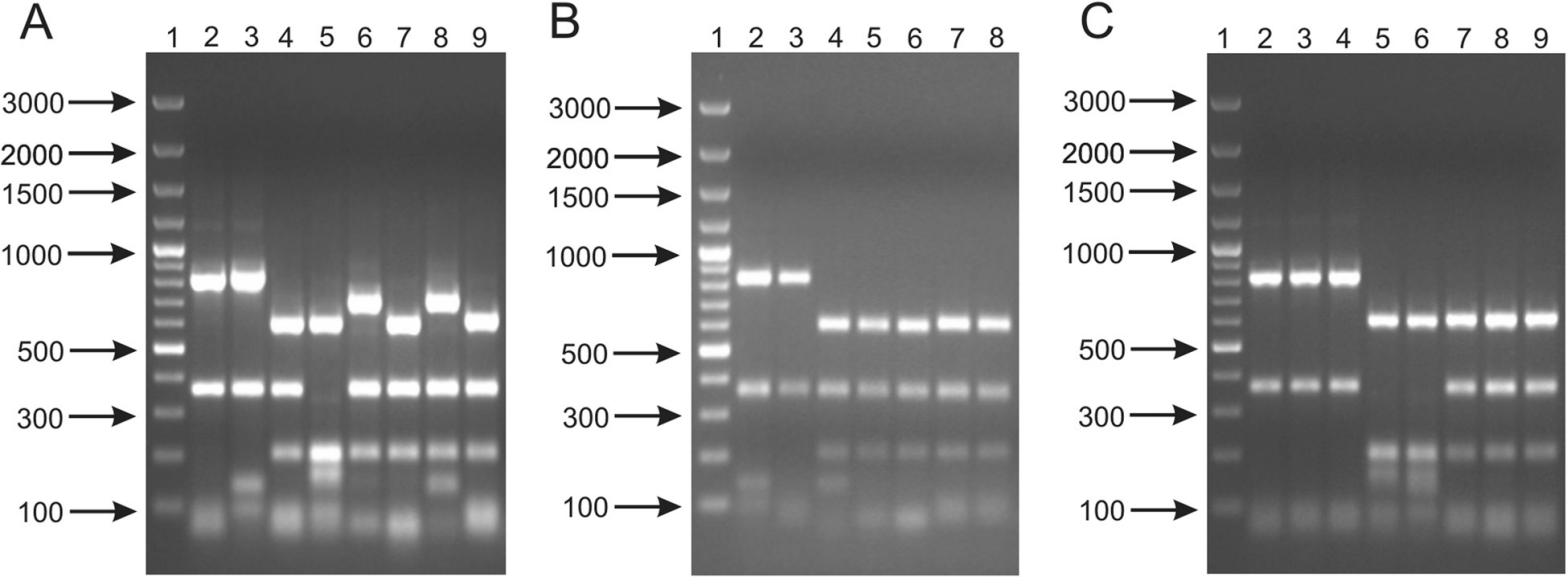
ARDRA patterns generated from restriction analysis of genus-specific amplicon (1350 bp) of 17 bifidobacterial strains using AluI restrictase. Analysis of the discriminatory power of the procedure applied was performed at a species level (**a**) - 1, DNA molecular marker; 2, *B. adolescentis* DSM 20087; 3, *B. animalis* NRRL B-41406; 4, *B. bifidum* DSM 204564; 5, *B. breve* DSM 20091; 6, *B. catenulatum* DSM 20224; 7, *B. longum* NRRL B-41409; 8, *B. pseudocatenulatum* DSM 20439; 9, *B. pseudolongum* DSM 20099; at a subspecies level (**b**) - 1, DNA molecular marker; 2, *B. animalis* subsp. *animalis* NRRL B-41406; 3, *B. animalis* subsp. *lactis* NRRL B-41405; 4, *B. longum* subsp. *infantis* ATCC 15697; 5, *B. longum* subsp. *longum* NRRL B-41409; 5, *B. longum* subsp. *suis* NRRL B-41407; 6, *B. pseudolongum* subsp. *pseudolongum* DSM 20099; 7, *B. pseudolongum* subsp. *globosum* DSM 20092; and at a strain level (**c**) - 1, DNA molecular marker; 2, *B. adolescentis* DSM 20087; 3, *B. adolescentis* DSM 20083; 4, *B. adolescentis* 20086; 5, *B. breve* DSM 20091; 6, *B. breve* NRRL B-41408; 7*, B. pseudolongum* DSM 20099; 8, *B. pseudolongum* 20094; 9, *B. pseudolongum* DSM 20095

In this work, genus-specific PCR products were digested by AluI, BsuRI and HinfI to enable rapid discrimination of selected *Bifidobacterium* strains at the species, subspecies and strain level. The results showed rather low discriminatory power of this procedure. It was not possible to clearly distinguish the analyzed *Bifidobacterium* strains even at the species level. For instance, *B. breve* had the same restriction pattern as *B. longum* and *B. pseudolongum* when Alu I restriction enzyme was used (Fig. 1a). Similarly, BsuRI and HinfI digestion of the amplicons resulted in identical restriction profiles for *B. adolescentis* and *B. bifidum* as well as for *B. animalis*, *B. longum* and *B. pseudolongum* (Additional file 1: Figure S1A and Additional file 2: Figure S2A). In addition, *B. catenulatum* could not be differentiated from *B. pseudocatenulatum* using BsuRI and HinfI. Although some researchers demonstrated that ARDRA is a reliable and reproducible molecular tool to determine bifidobacterial species [32, 34], in our opinion it is very difficult to design a universal procedure providing unique restriction patterns for all closely related species, especially using only one restriction enzyme. This is due to the high sequence similarity (87.7–99.5 %) of the 16S rRNA gene among bifidobacterial species [16]. Therefore, restriction analysis of other genes, e.g., *hsp60* [35] or *bsh* [36] seems to be a more suitable route for the identification of *Bifidobacterium* species. On the other hand, our results and previous reports showed that in some cases ARDRA was a very reproducible molecular approach for distinguishing between certain subspecies of *Bifidobacterium* [37]. In our experiments, unquestionable separation of *B. longum* subsp. *infantis* from two other subspecies of *B. longum* (*B. longum* subsp. *longum* and *B. longum* subsp. *suis*) was achieved using AluI restriction enzyme (Fig. 1b). *B. longum* subsp. *infantis* digestion resulted in four products of approximately 600, 370, 210 and 130 bp. *B. longum* subsp. *longum* and *B. longum* subsp. *suis* displayed similar digestion patterns, however, the shortest fragments were approximately 85 bp in size. AluI restriction patterns also differentiated *B. animalis* subsp. *animalis* and *B. animalis* subsp. *lactis*. In case of *B. animalis* subsp. *animalis*, AluI digestion generated four fragments (approx. 845, 370, 130 and 100 bp), whereas *B. animalis* subsp. *lactis* had only three detectable bands of approximately 845, 370 and 95 bp. Moreover, the analysis of restriction patterns of the 16S rRNA gene generated for *B. adolescentis* DSM 20087 allowed to discriminate this strain from two other *B. adolescentis* isolates (Additional file 1: Figure S1C and Additional file 2: Figure S2C). Interestingly, in the case of Hinf1 restriction pattern, one additional band of approximately 320 bp was clearly visible for *B. adolescentis* DSM 20087 (Additional file 2: Figure S2C). This result was presumably obtained, because some alleles of the 16S rRNA gene may have an additional HinfI restriction site in this microorganism. A similar phenomenon was previously described by Heyndrickx et al. [30].

In summary, many authors describe restriction length polymorphism of the 16S rRNA gene as a reliable and reproducible approach for taxonomic and phylogenetic analyses of the genus *Bifidobacterium*; moreover, the repeated and accurate differentiation of various species and even subspecies of *Bifidobacterium* is well documented. However, due to the high 16S rRNA sequence similarity, this molecular tool also has its limitations. In some cases, e. g., when closely-related taxa are analyzed, the discriminatory power of this technique is too low, and thus more than one restriction enzyme needs to be used. Hence, despite its high reproducibility, this procedure appears to be more laborious and time-consuming compared to other PCR-based methods.

### Randomly amplified polymorphic DNA (RAPD-PCR)

Ample works indicate that the beneficial properties of probiotic bacteria are strain-dependent. Therefore, the application of rapid and reliable typing methods for precise identification of specific strains is necessary. In 1990, Williams et al. [38] described a simple and rapid method called randomly amplified polymorphic DNA (RAPD), which is frequently used to discriminate between bacterial species, and to some extent, also between strains within the same species [12, 38, 39].

**Fig. 2:**
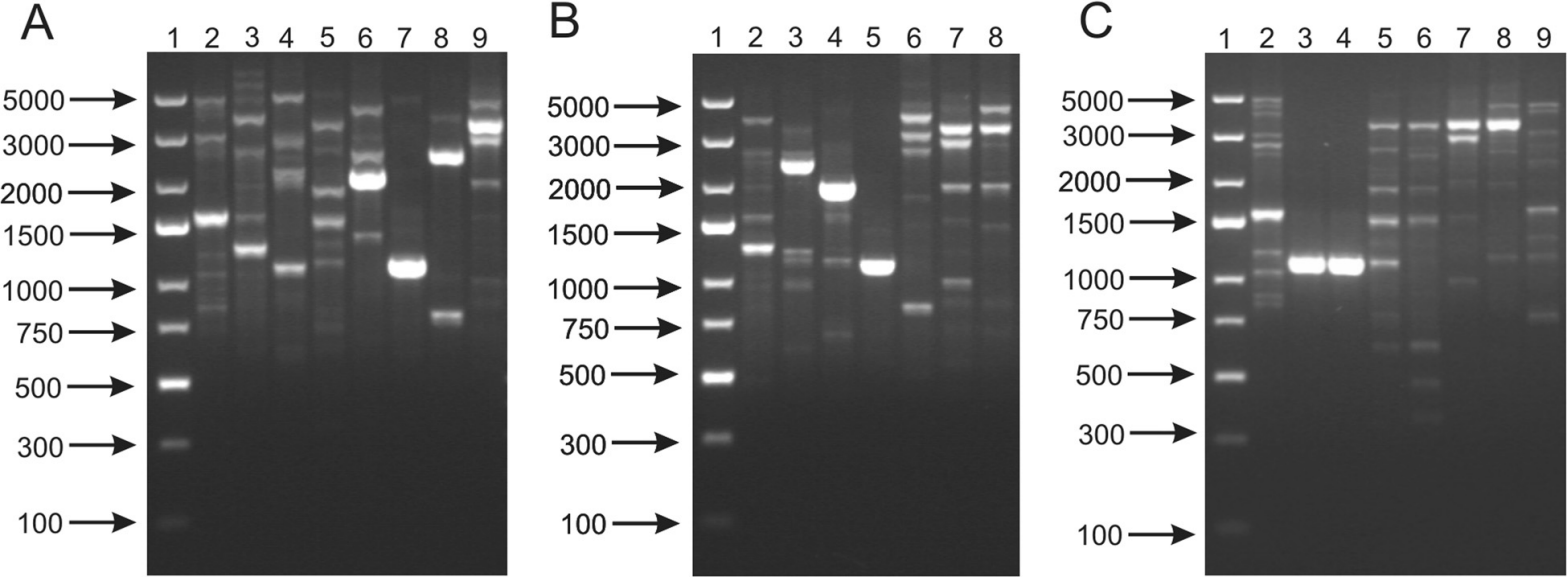
Randomly amplified polymorphic DNA (RAPD)-PCR patterns obtained with PER1 primer for 17 bifidobacterial strains. Analysis of the discriminatory power of the procedure applied was performed at a species level (**a**) - 1, DNA molecular marker; 2, *B. adolescentis* DSM 20087; 3, *B. animalis* NRRL B-41406; 4, *B. bifidum* DSM 204564; 5, *B. breve* DSM 20091; 6, *B. catenulatum* DSM 20224; 7, *B. longum* NRRL B-41409; 8, *B. pseudocatenulatum* DSM 20439; 9, *B. pseudolongum* DSM 20099; at a subspecies level (**b**) - 1, DNA molecular marker; 2, *B. animalis* subsp. *animalis* NRRL B-41406; 3, *B. animalis* subsp. *lactis* NRRL B-41405; 4, *B. longum* subsp. *infantis* ATCC 15697; 5, *B. longum* subsp. *longum* NRRL B-41409; 5, *B. longum* subsp. *suis* NRRL B-41407; 6, *B. pseudolongum* subsp. *pseudolongum* DSM 20099; 7, *B. pseudolongum* subsp. *globosum* DSM 20092; and at a strain level (**c**) - 1, DNA molecular marker; 2, *B. adolescentis* DSM 20087; 3, *B. adolescentis* DSM 20083; 4, *B. adolescentis* 20086; 5, *B. breve* DSM 20091; 6, *B. breve* NRRL B-41408; 7*, B. pseudolongum* DSM 20099; 8, *B. pseudolongum* 20094; 9, *B. pseudolongum* DSM 20095

In the present study, two previously described RAPD primers, PER1 [25] and CORR1 [26], were used to differentiate 17 bifidobacterial strains. Our results showed that all analyzed species and subspecies of *Bifidobacterium* strains had distinct fingerprinting patterns. Clear distinction between bifidobacterial species and subspecies was possible in reactions with both PCR oligonucleotides (Fig. 2 and Additional file 3: Figure S3). These results are consistent with previous works of Vincent et al. [40] and Krizova et al. [17] who proved that RAPD was an appropriate molecular tool for the identification of bifidobacteria at the species and subspecies level. Moreover, our experiments revealed that RAPD has a potential to differentiate bifidobacteria at the strain level. PCRs performed with the PER1 primer allowed to accurately discriminate between *B. breve* DSM 20091 and *B. breve* NRRL B-41408, and also three *B. pseudolongum* strains (DSM 20094, DSM 20095 and DSM 20099). Two *B. adolescentis* strains (DSM 20083 and DSM 20086) could not be determined in these experimental conditions, as only a single band was observed for these strains after electrophoretic separation. In the case of RAPD reaction with the CORR1oligonucleotide, DNA patterns of some of the strains had a low intensity and, therefore, were difficult to analyze. Such results suggest that additional reactions with other random primers should be performed to successfully distinguish these strains of bifidobacteria.

Some researchers claim that fingerprinting methods have the disadvantage of low reproducibility and require precise standardization of PCR conditions in comparison to ARDRA. The use of different polymerases, primer to template ratios, magnesium concentrations, different annealing temperatures and also different thermal cyclers may lead to variations in DNA patterns obtained in different laboratories [13, 35, 39]. In turn, Vincent et al. [40] indicated that when standardized PCR protocol was applied, reproducible results were obtained for 18 *Bifidobacterium* strains using DNA samples from two separate extractions. Additionally, RAPD reactions conducted in two different thermal cyclers proved the reproducibility of this technique [40]. Therefore, we conclude that this method is an effective tool for typing of *Bifidobacterium* strains, especially when standardized PCR reactions with several random primers are applied.

### Repetitive element sequence-based polymerase chain reaction (rep-PCR)

Rep-PCR is a genomic DNA fingerprinting method based on the widespread distribution of evolutionarily conserved repetitive DNA elements such as BOX, ERIC, REP and (GTG)_5_ [18, 41]. Previous publications showed that rep-PCR may be used for rapid and easy differentiation of both Gram-negative and Gram-positive microorganisms. This method was also described as a powerful molecular tool for both identification and typing of bifidobacteria as well as species composition analysis of complex bifidobacterial cultures isolated from dairy products and infant formulas [12, 13, 42]. A study conducted by Masco et al. [18] demonstrated that BOX-PCR and (GTG)_5_-PCR were the most suitable rep-PCR procedures for accurate identification of various *Bifidobacterium* strains. Based on this work, BOXA1R and (GTG)_5_ primers were selected for our experiments.

**Fig. 3:**
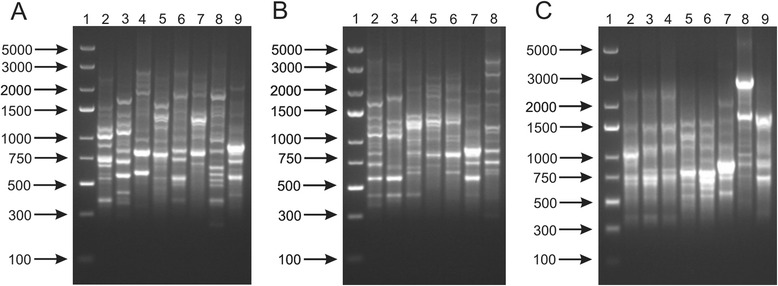
(GTG)_5_-PCR patterns of 17 strains belonging to the genus *Bifidobacterium*. Analysis of the discriminatory power of the procedure applied was performed at a species level (**a**) - 1, DNA molecular marker; 2, *B. adolescentis* DSM 20087; 3, *B. animalis* NRRL B-41406; 4, *B. bifidum* DSM 204564; 5, *B. breve* DSM 20091; 6, *B. catenulatum* DSM 20224; 7, *B. longum* NRRL B-41409; 8, *B. pseudocatenulatum* DSM 20439; 9, *B. pseudolongum* DSM 20099; at a subspecies level (**b**) - 1, DNA molecular marker; 2, *B. animalis* subsp. *animalis* NRRL B-41406; 3, *B. animalis* subsp. *lactis* NRRL B-41405; 4, *B. longum* subsp. *infantis* ATCC 15697; 5, *B. longum* subsp. *longum* NRRL B-41409; 5, *B. longum* subsp. *suis* NRRL B-41407; 6, *B. pseudolongum* subsp. *pseudolongum* DSM 20099; 7, *B. pseudolongum* subsp. *globosum* DSM 20092; and at a strain level (**c**) - 1, DNA molecular marker; 2, *B. adolescentis* DSM 20087; 3, *B. adolescentis* DSM 20083; 4, *B. adolescentis* 20086; 5, *B. breve* DSM 20091; 6, *B. breve* NRRL B-41408; 7*, B. pseudolongum* DSM 20099; 8, *B. pseudolongum* 20094; 9, *B. pseudolongum* DSM 20095

**Fig. 4:**
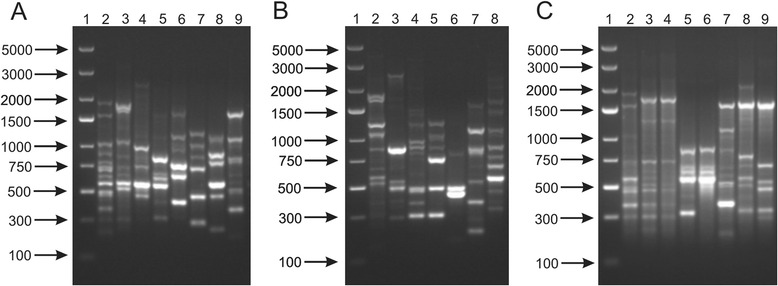
BOX-PCR DNA profiles obtained for *Bifidobacterium* strains used in this work. Analysis of the discriminatory power of this procedure was performed at a species level (**a**) - 1, DNA molecular marker; 2, *B. adolescentis* DSM 20087; 3, *B. animalis* NRRL B-41406; 4, *B. bifidum* DSM 204564; 5, *B. breve* DSM 20091; 6, *B. catenulatum* DSM 20224; 7, *B. longum* NRRL B-41409; 8, *B. pseudocatenulatum* DSM 20439; 9, *B. pseudolongum* DSM 20099; at a subspecies level (**b**) - 1, DNA molecular marker; 2, *B. animalis* subsp. *animalis* NRRL B-41406; 3, *B. animalis* subsp. *lactis* NRRL B-41405; 4, *B. longum* subsp. *infantis* ATCC 15697; 5, *B. longum* subsp. *longum* NRRL B-41409; 5, *B. longum* subsp. *suis* NRRL B-41407; 6, *B. pseudolongum* subsp. *pseudolongum* DSM 20099; 7, *B. pseudolongum* subsp. *globosum* DSM 20092; and at a strain level (**c**) - 1, DNA molecular marker; 2, *B. adolescentis* DSM 20087; 3, *B. adolescentis* DSM 20083; 4, *B. adolescentis* 20086; 5, *B. breve* DSM 20091; 6, *B. breve* NRRL B-41408; 7*, B. pseudolongum* DSM 20099; 8, *B. pseudolongum* 20094; 9, *B. pseudolongum* DSM 20095

In this analysis, different DNA profiles were observed for all *Bifidobacterium* species. Similarly to previous observations of Gomez Zavaglia et al. [43], Masco et al. [18] and Krizova et al. [17], it was possible to differentiate even closely related species such as *B. longum* and *B. breve* or *B. catenulatum* and *B. pseudocatenulatum* in PCR reactions with (GTG)_5_ (Fig. 3a) and BOXA1R oligonucleotides (Fig. 4a). Furthermore, the resulting DNA patterns in both procedures were sufficient to successfully discriminate between the tested bifidobacteria at the subspecies level (Fig. 3b and 4b). In addition, pronounced differences were observed for bacterial strains of the same species. Similarly to RAPD patterns, it was difficult to differentiate unambiguously between two *B. adolescentis* strains (DSM 20083 and DSM 20086) using the BOXA1R primer. On the other hand, this method showed clear differences between two strains of *B. breve* and three strains belonging to *B. pseudolongum* subsp. *pseudolongum*. PCR reactions with the (GTG)_5_ primer resulted in similar, however, not identical profiles of *B. adolescentis* and *B. breve* bifidobacterial strains. As in the case of BOXA1R, (GTG)_5_ PCR also provided distinct fingerprinting patterns for all *B. pseudolongum* strains tested.

Some researchers indicated that similarly to other DNA fingerprinting methods, it was problematic to compare rep-PCR results obtained in different laboratories [44]. This dissimilarity is mainly due to the different PCR reaction composition as well as various electrophoresis conditions. In contrast, other publications reported high reproducibility of these methods (>90 %), arguing that the observed differences are usually due to the different signal intensity of a specific band rather than the lack or presence of additional bands [18, 45]. Nevertheless, based on our results, it is evident that this approach is a highly discriminatory, easy-to-handle and relatively low-cost procedure for rapid differentiation of bifidobacteria at the intra-species level. In comparison to RAPD-PCR, this technique has a similar discriminatory power, however, only one oligonucleotide is necessary in rep-PCR to obtain strain-specific DNA fingerprints. This is consistent with a previous work of Krizova et al. [17], who concluded that only a combination of several RAPD primers enabled good discrimination of the tested bifidobacteria. Therefore, from the practical perspective, when a high number of isolates needs to be identified, rep-PCR seems to be a more convenient and reliable tool for rapid differentiation of bifidobacteria.

### Analysis of whole-cell protein fingerprinting using SDS-PAGE

Many researchers reported that SDS-PAGE of whole-cell proteins was a useful molecular tool for identification and characterization of different microorganisms [29, 46, 47]. Moreover, this method turned out to be faster and more cost-effective than other genotypic methods, which are commonly applied in bacteria differentiation. It was also proved that this approach was particularly effective for discrimination and grouping of a large number of newly isolated strains [29].

**Fig. 5:**
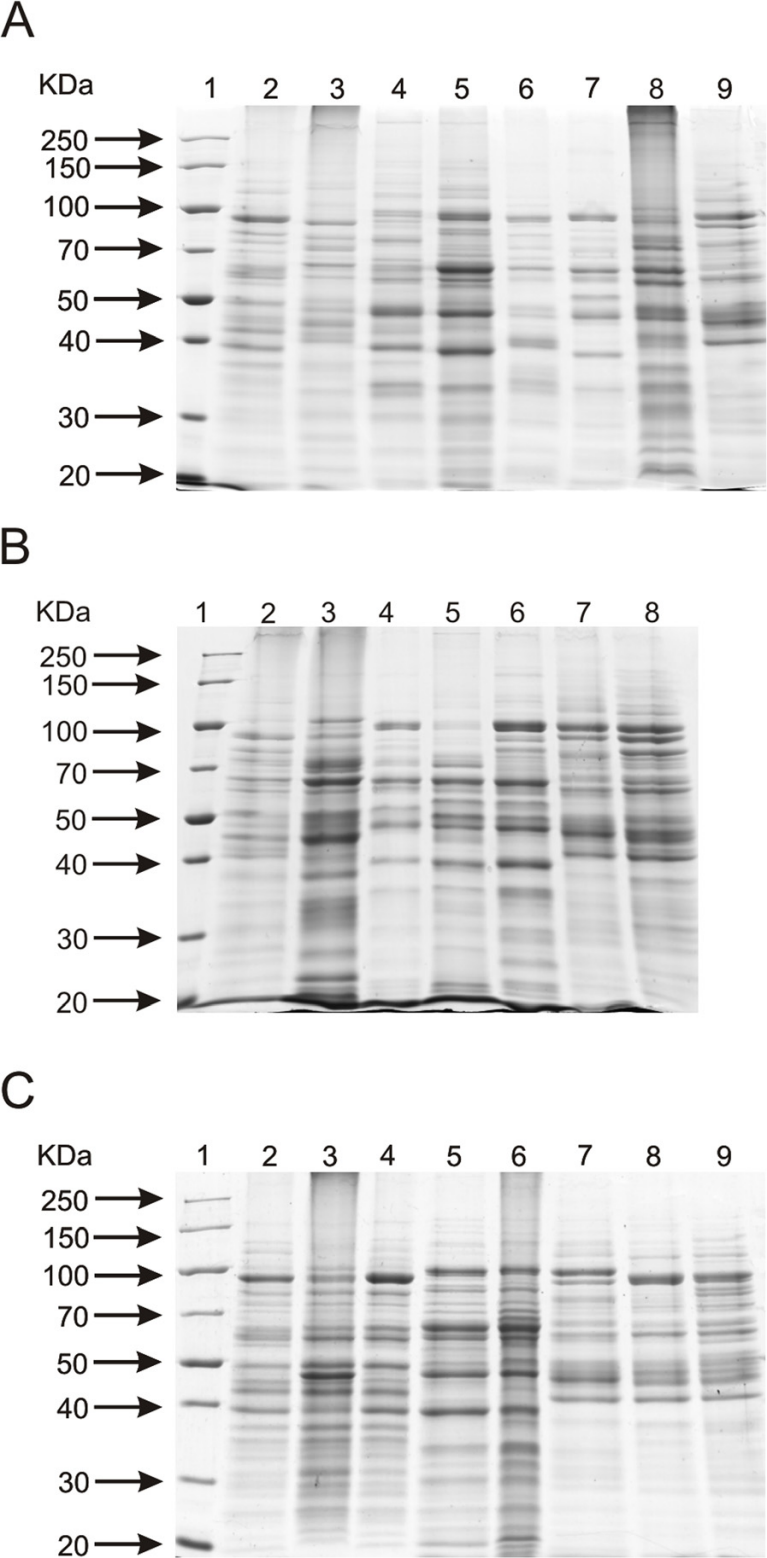
SDS-PAGE profiles of whole cell proteins obtained for all tested bifidobacteria. Analysis of the discriminatory power of this technique was performed at a species level (**a**) - 1, protein molecular weight marker; 2, *B. adolescentis* DSM 20087; 3, *B. animalis* NRRL B-41406; 4, *B. bifidum* DSM 204564; 5, *B. breve* DSM 20091; 6, *B. catenulatum* DSM 20224; 7, *B. longum* NRRL B-41409; 8, *B. pseudocatenulatum* DSM 20439; 9, *B. pseudolongum* DSM 20099; at a subspecies level (**b**) – 1, protein molecular weight marker; 2, *B. animalis* subsp. *animalis* NRRL B-41406; 3, *B. animalis* subsp. *lactis* NRRL B-41405; 4, *B. longum* subsp. *infantis* ATCC 15697; 5, *B. longum* subsp. *longum* NRRL B-41409; 5, *B. longum* subsp. *suis* NRRL B-41407; 6, *B. pseudolongum* subsp. *pseudolongum* DSM 20099; 7, *B. pseudolongum* subsp. *globosum* DSM 20092; and at a strain level (**c**) - 1, protein molecular weight marker; 2, *B. adolescentis* DSM 20087; 3, *B. adolescentis* DSM 20083; 4, *B. adolescentis* 20086; 5, *B. breve* DSM 20091; 6, *B. breve* NRRL B-41408; 7*, B. pseudolongum* DSM 20099; 8, *B. pseudolongum* 20094; 9, *B. pseudolongum* DSM 20095

In the present work, whole-cell proteins were analyzed from 17 strains of the genus *Bifidobacterium*. The analysis of protein profiles obtained after electrophoretic separation confirmed the effectiveness of this method for bifidobacteria differentiation [48, 49]. The electrophoretic protein fingerprints of the examined strains showed the presence of numerous bands with molecular mass ranging from 20 to 250 kDa. As expected, clear visual differences were observed in protein profiles among the tested bifidobacteria both at the species and subspecies level (Fig. 5a and b). It is consistent with previous reports, in which the SDS-PAGE fingerprinting was described as a useful and reliable tool for identifying species and subspecies of lactic acid bacteria [29, 49, 50]. Nonetheless, because of the high number of protein bands generated as well as different band intensities, this procedure seems to be less suitable when closely related strains are analyzed (Fig. 5c). These observations confirmed previous findings that the SDS-PAGE fingerprinting provides a lower taxonomic resolution compared to genotypic fingerprinting, where gel interpretation is easier in comparison to the analysis of whole-cell proteins [19]. However, this method turns out to be a valuable alternative for genotypic techniques, as it allows for fast screening of a large number of strains, especially for comparative purposes or as part of polyphasic taxonomic studies [29, 46].

### Differentiation of newly isolated bifidobacteria using (GTG)_5_-PCR and BOX-PCR fingerprinting

**Fig. 6:**
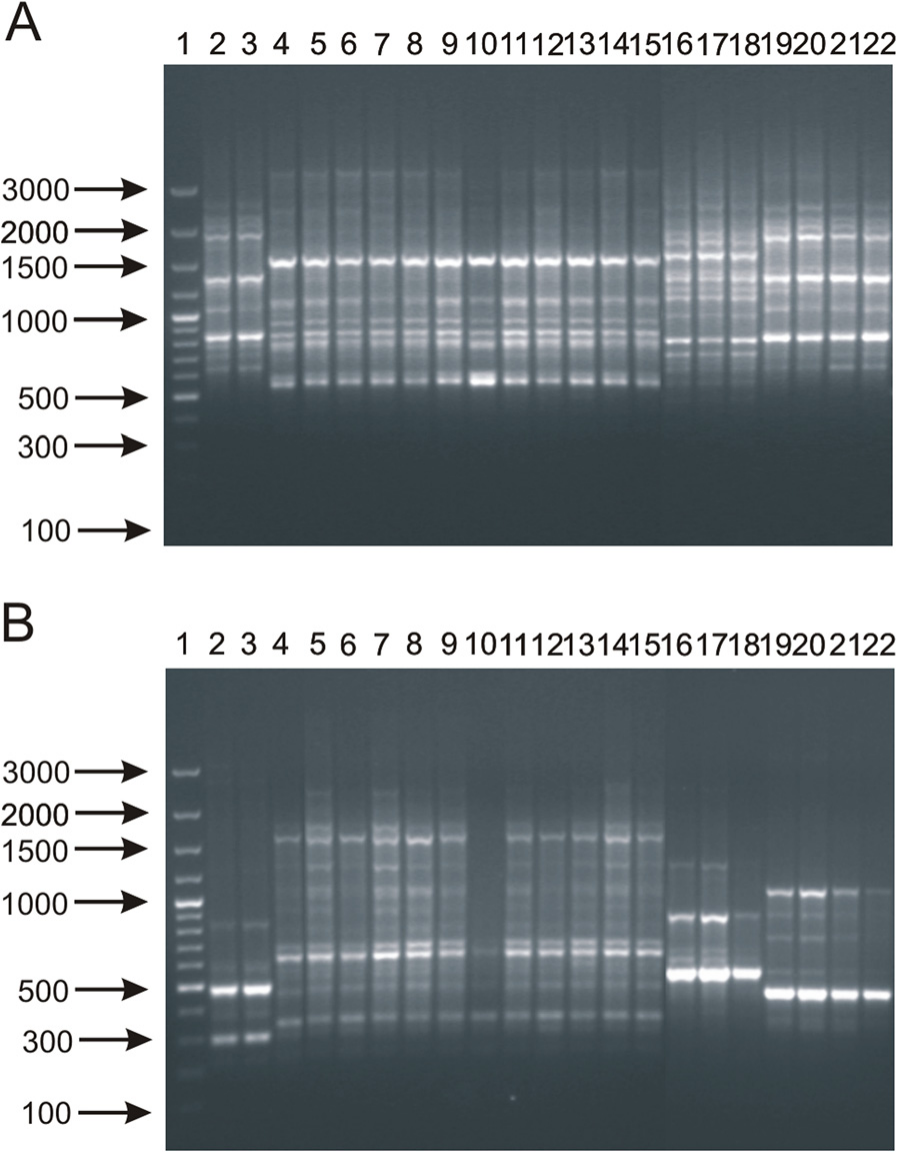
Differentiation of 21 bifidobacterial strains isolated from child feces using rep-PCR procedures. DNA profiles were determined in PCR reaction with (GTG)_5_ primer (**a**) and BOX1R oligonucleotide (**b**). Lane: 1, DNA molecular marker, 2, *Bifdobacterium* NK1.2; 3, NK2.2; 4, NK6.1; 5, NK7.2; 6, NK8.1; 7, NK9.1; 8, NK10.2; 9, NK11.1; 10, NK12; 11, NK13; 12, NK14; 13, NK15; 14, NK16; 15, NK17; 16, MP1; 17, MP5; 18, MP6; 19, WP3; 20, WP4; 21, WP7; 22, WP8

Based on the comparison of different procedures, rep-PCR methods were selected for rapid differentiation of 21 *Bifidobacterium* strains isolated from infant feces. Firstly, to confirm that the newly isolated microorganisms belong to the genus *Bifidobacterium*, partial 16S rRNA genes were amplified using genus-specific primers [24]. Specific amplification products (about 1350 bp) were obtained for 21 isolates tested (data not shown). Next, the differentiation of these isolates was conducted using BOX-PCR and (GTG)_5_-PCR. The BOXA1R primer allowed a slightly better discrimination of the tested isolates (Fig. 6a and b). The results are consistent with the studies described by Masco et al. [18] and Krizova et al. [17] who showed that the patterns generated in reaction with the BOXA1R primer displayed a higher inter-species heterogeneity compared to DNA profiles obtained with (GTG)_5_.

## Conclusions

In conclusion, this work evaluated the discriminatory power of four molecular methods, which are extensively used for fast differentiation of various microorganisms. Our experiments confirmed that the restriction analysis of the amplified 16S rRNA gene is a reliable and highly reproducible approach for typing *Bifidobacterium* strains. Nevertheless, the discriminatory power of this method is rather limited and strongly depends on restriction enzymes used and the length of amplicons. In comparison to ARDRA, genotypic fingerprinting procedures (RAPD and rep-PCR) seem to be less reproducible and less comparable between different laboratories. Despite this, they allow to differentiate the strains even at the intra-species level, and therefore, they are more suitable for rapid discrimination of a high number of newly isolated microorganisms. It was also confirmed that RAPD and rep-PCR had similar discriminatory powers, though, in some instances, more than one oligonucleotide was needed in case of differentiation with random primers. The last method tested was an electrophoretic analysis of whole-cell proteins. Due to its high discriminatory power and relatively inexpensive experiments, this procedure may be used as an alternative to PCR-based methods.

In summary, the tested methods are currently the most popular approaches for differentiation and typing of bacteria. However, as shown in this study, some of them have too low discriminatory power to exclude the possibility of multiple isolation of the same strain. In this study, as in the previous report of Masco et al. [18], the BOX-PCR procedure proved to be the most effective and convenient molecular technique in differentiating *Bifidobacterium* strains at all taxonomic levels. However, it is still very important, to improve the existing typing methods and develop new procedures for rapid and reliable bacteria identification, especially at the intra-species level.

## Abbreviations

ARDRA, amplified ribosomal DNA restriction analysis; BOX-PCR, PCR based on primers targeting the highly conserved repetitive DNA sequences of BOXA subunit of the BOX element; bp, base pair; ERIC-PCR, enterobacterial repetitive intergenic consensus PCR; kDa, kilodalton; PCR, polymerase chain reaction; PFGE, pulsed-field gel electrophoresis; RAPD, random amplified polymorphic DNA; rep-PCR, repetitive sequence-based PCR; RFLP, restriction fragment length polymorphism; SDS-PAGE, sodium dodecyl sulfate polyacrylamide gel electrophoresis

## Additional files

Additional file 1: Figure S1. ARDRA patterns generated from restriction analysis of genus-specific amplicon (1350 bp) of 17 bifidobacterial strains using BsuRI restrictase. Analysis of the discriminatory power of the procedure applied was performed at a species level (A) - 1, DNA molecular marker; 2, *B. adolescentis* DSM 20087; 3, *B. animalis* NRRL B-41406; 4, *B. bifidum* DSM 204564; 5, *B. breve* DSM 20091; 6, *B. catenulatum* DSM 20224; 7, *B. longum* NRRL B-41409; 8, *B. pseudocatenulatum* DSM 20439; 9, *B. pseudolongum* DSM 20099; at a subspecies level (B) - 1, DNA molecular marker; 2, *B. animalis* subsp. *animalis* NRRL B-41406; 3, *B. animalis* subsp. *lactis* NRRL B-41405; 4, *B. longum* subsp. *infantis* ATCC 15697; 5, *B. longum* subsp. *longum* NRRL B-41409; 5, *B. longum* subsp. *suis* NRRL B-41407; 6, *B. pseudolongum* subsp. *pseudolongum* DSM 20099; 7, *B. pseudolongum* subsp. *globosum* DSM 20092; and at a strain level (C) - 1, DNA molecular marker; 2, *B. adolescentis* DSM 20087; 3, *B. adolescentis* DSM 20083; 4, *B. adolescentis* 20086; 5, *B. breve* DSM 20091; 6, *B. breve* NRRL B-41408; 7*, B. pseudolongum* DSM 20099; 8, *B. pseudolongum* 20094; 9, *B. pseudolongum* DSM 20095. (TIF 567 kb) Additional file 2: Figure S2. ARDRA patterns generated from restriction analysis of genus-specific amplicon (1350 bp) of 17 bifidobacterial strains using HinfI restrictase. Analysis of the discriminatory power of the procedure used was performed at a species level (A) - 1, DNA molecular marker; 2, *B. adolescentis* DSM 20087; 3, *B. animalis* NRRL B-41406; 4, *B. bifidum* DSM 204564; 5, *B. breve* DSM 20091; 6, *B. catenulatum* DSM 20224; 7, *B. longum* NRRL B-41409; 8, *B. pseudocatenulatum* DSM 20439; 9, *B. pseudolongum* DSM 20099; at a subspecies level (B) - 1, DNA molecular marker; 2, *B. animalis* subsp. *animalis* NRRL B-41406; 3, *B. animalis* subsp. *lactis* NRRL B-41405; 4, *B. longum* subsp. *infantis* ATCC 15697; 5, *B. longum* subsp. *longum* NRRL B-41409; 5, *B. longum* subsp. *suis* NRRL B-41407; 6, *B. pseudolongum* subsp. *pseudolongum* DSM 20099; 7, *B. pseudolongum* subsp. *globosum* DSM 20092; and at a strain level (C) - 1, DNA molecular marker; 2, *B. adolescentis* DSM 20087; 3, *B. adolescentis* DSM 20083; 4, *B. adolescentis* 20086; 5, *B. breve* DSM 20091; 6, *B. breve* NRRL B-41408; 7*, B. pseudolongum* DSM 20099; 8, *B. pseudolongum* 20094; 9, *B. pseudolongum* DSM 20095. (TIF 589 kb) Additional file 3: Figure S3. Randomly amplified polymorphic DNA (RAPD)-PCR patterns obtained with CORR1 primer for 17 bifidobacterial strains. Analysis of the discriminatory power of the procedure applied was performed at a species level (A) - 1, DNA molecular marker; 2, *B. adolescentis* DSM 20087; 3, *B. animalis* NRRL B-41406; 4, *B. bifidum* DSM 204564; 5, *B. breve* DSM 20091; 6, *B. catenulatum* DSM 20224; 7, *B. longum* NRRL B-41409; 8, *B. pseudocatenulatum* DSM 20439; 9, *B. pseudolongum* DSM 20099; at a subspecies level (B) - 1, DNA molecular marker; 2, *B. animalis* subsp. *animalis* NRRL B-41406; 3, *B. animalis* subsp. *lactis* NRRL B-41405; 4, *B. longum* subsp. *infantis* ATCC 15697; 5, *B. longum* subsp. *longum* NRRL B-41409; 5, *B. longum* subsp. *suis* NRRL B-41407; 6, *B. pseudolongum* subsp. *pseudolongum* DSM 20099; 7, *B. pseudolongum* subsp. *globosum* DSM 20092; and at a strain level (C) - 1, DNA molecular marker; 2, *B. adolescentis* DSM 20087; 3, *B. adolescentis* DSM 20083; 4, *B. adolescentis* 20086; 5, *B. breve* DSM 20091; 6, *B. breve* NRRL B-41408; 7*, B. pseudolongum* DSM 20099; 8, *B. pseudolongum* 20094; 9, *B. pseudolongum* DSM 20095. (TIF 555 kb)
