# Supplementary figures and images for: Comparison of various molecular methods for rapid differentiation of intestinal bifidobacteria at the species, subspecies and strain level
Source: BMC Microbiol. 2016 Jul 22;16:159. doi: 10.1186/s12866-016-0779-3 (PMC4957357; doi:10.1186/s12866-016-0779-3)

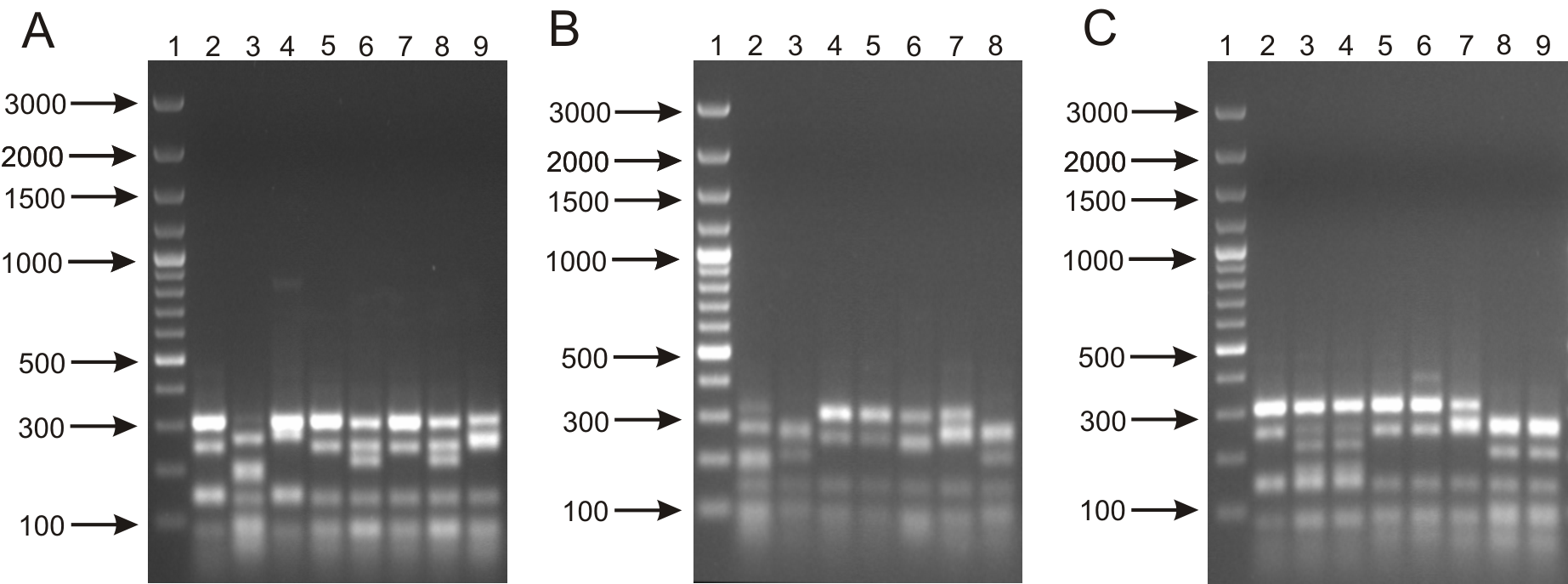

Supplement: Additional file 1: Figure S1. — ARDRA patterns generated from restriction analysis of genus-specific amplicon (1350 bp) of 17 bifidobacterial strains using BsuRI restrictase. Analysis of the discriminatory power of the procedure applied was performed at a species level (A) - 1, DNA molecular marker; 2, B. adolescentis DSM 20087; 3, B. animalis NRRL B-41406; 4, B. bifidum DSM 204564; 5, B. breve DSM 20091; 6, B. catenulatum DSM 20224; 7, B. longum NRRL B-41409; 8, B. pseudocatenulatum DSM 20439; 9, B. pseudolongum DSM 20099; at a subspecies level (B) - 1, DNA molecular marker; 2, B. animalis subsp. animalis NRRL B-41406; 3, B. animalis subsp. lactis NRRL B-41405; 4, B. longum subsp. infantis ATCC 15697; 5, B. longum subsp. longum NRRL B-41409; 5, B. longum subsp. suis NRRL B-41407; 6, B. pseudolongum subsp. pseudolongum DSM 20099; 7, B. pseudolongum subsp. globosum DSM 20092; and at a strain level (C) - 1, DNA molecular marker; 2, B. adolescentis DSM 20087; 3, B. adolescentis DSM 20083; 4, B. adolescentis 20086; 5, B. breve DSM 20091; 6, B. breve NRRL B-41408; 7, B. pseudolongum DSM 20099; 8, B. pseudolongum 20094; 9, B. pseudolongum DSM 20095. (TIF 567 kb) [file 12866_2016_779_MOESM1_ESM.tif]

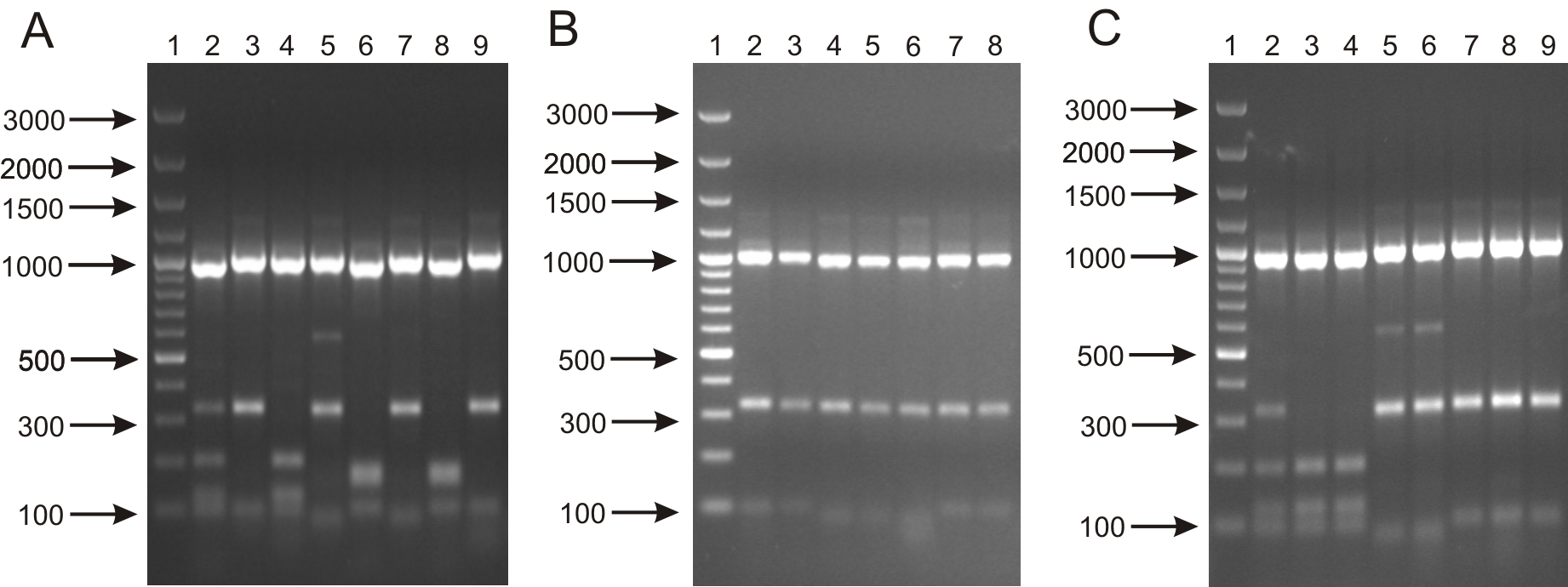

Supplement: Additional file 2: Figure S2. — ARDRA patterns generated from restriction analysis of genus-specific amplicon (1350 bp) of 17 bifidobacterial strains using HinfI restrictase. Analysis of the discriminatory power of the procedure used was performed at a species level (A) - 1, DNA molecular marker; 2, B. adolescentis DSM 20087; 3, B. animalis NRRL B-41406; 4, B. bifidum DSM 204564; 5, B. breve DSM 20091; 6, B. catenulatum DSM 20224; 7, B. longum NRRL B-41409; 8, B. pseudocatenulatum DSM 20439; 9, B. pseudolongum DSM 20099; at a subspecies level (B) - 1, DNA molecular marker; 2, B. animalis subsp. animalis NRRL B-41406; 3, B. animalis subsp. lactis NRRL B-41405; 4, B. longum subsp. infantis ATCC 15697; 5, B. longum subsp. longum NRRL B-41409; 5, B. longum subsp. suis NRRL B-41407; 6, B. pseudolongum subsp. pseudolongum DSM 20099; 7, B. pseudolongum subsp. globosum DSM 20092; and at a strain level (C) - 1, DNA molecular marker; 2, B. adolescentis DSM 20087; 3, B. adolescentis DSM 20083; 4, B. adolescentis 20086; 5, B. breve DSM 20091; 6, B. breve NRRL B-41408; 7, B. pseudolongum DSM 20099; 8, B. pseudolongum 20094; 9, B. pseudolongum DSM 20095. (TIF 589 kb) [file 12866_2016_779_MOESM2_ESM.tif]

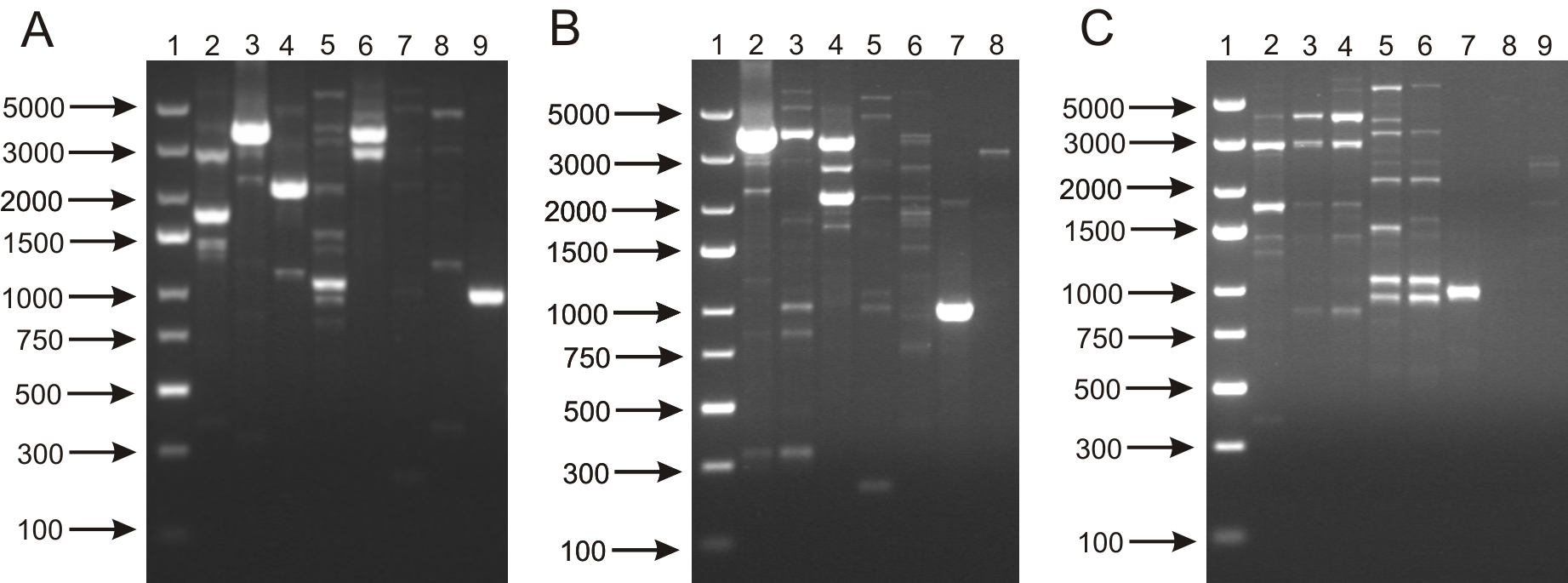

Supplement: Additional file 3: Figure S3. — Randomly amplified polymorphic DNA (RAPD)-PCR patterns obtained with CORR1 primer for 17 bifidobacterial strains. Analysis of the discriminatory power of the procedure applied was performed at a species level (A) - 1, DNA molecular marker; 2, B. adolescentis DSM 20087; 3, B. animalis NRRL B-41406; 4, B. bifidum DSM 204564; 5, B. breve DSM 20091; 6, B. catenulatum DSM 20224; 7, B. longum NRRL B-41409; 8, B. pseudocatenulatum DSM 20439; 9, B. pseudolongum DSM 20099; at a subspecies level (B) - 1, DNA molecular marker; 2, B. animalis subsp. animalis NRRL B-41406; 3, B. animalis subsp. lactis NRRL B-41405; 4, B. longum subsp. infantis ATCC 15697; 5, B. longum subsp. longum NRRL B-41409; 5, B. longum subsp. suis NRRL B-41407; 6, B. pseudolongum subsp. pseudolongum DSM 20099; 7, B. pseudolongum subsp. globosum DSM 20092; and at a strain level (C) - 1, DNA molecular marker; 2, B. adolescentis DSM 20087; 3, B. adolescentis DSM 20083; 4, B. adolescentis 20086; 5, B. breve DSM 20091; 6, B. breve NRRL B-41408; 7, B. pseudolongum DSM 20099; 8, B. pseudolongum 20094; 9, B. pseudolongum DSM 20095. (TIF 555 kb) [file 12866_2016_779_MOESM3_ESM.tif]
